# Protein and RNA dynamical fingerprinting

**DOI:** 10.1038/s41467-019-08926-3

**Published:** 2019-03-04

**Authors:** Katherine A. Niessen, Mengyang Xu, Deepu K. George, Michael C. Chen, Adrian R. Ferré-D’Amaré, Edward H. Snell, Vivian Cody, James Pace, Marius Schmidt, Andrea G. Markelz

**Affiliations:** 1 Department of Physics, University at Buffalo, SUNY, Buffalo, NY USA; 2 National Heart, Lung and Blood Institute, Bethesda, MD USA; 3 Hauptman-Woodward Medical Research Institute & Department of Structural Biology, University at Buffalo, SUNY, Buffalo, NY USA; 4 Department of Physics, University of Wisconsin, Milwaukee, WI USA

## Abstract

Protein structural vibrations impact biology by steering the structure to functional intermediate states; enhancing tunneling events; and optimizing energy transfer. Strong water absorption and a broad continuous vibrational density of states have prevented optical identification of these vibrations. Recently spectroscopic signatures that change with functional state were measured using anisotropic terahertz microscopy. The technique however has complex sample positioning requirements and long measurement times, limiting access for the biomolecular community. Here we demonstrate that a simplified system increases spectroscopic structure to dynamically fingerprint biomacromolecules with a factor of 6 reduction in data acquisition time. Using this technique, polarization varying anisotropy terahertz microscopy, we show sensitivity to inhibitor binding and unique vibrational spectra for several proteins and an RNA G-quadruplex. The technique’s sensitivity to anisotropic absorbance and birefringence provides rapid assessment of macromolecular dynamics that impact biology.

## Abstract (web summary)

The characterization of biomacromolecule structural vibrations has been impeded by a broad continuous vibrational density of states obscuring molecule specific vibrations. A terahertz microscopy system using polarization control produces signatures to dynamically fingerprint proteins and a RNA G-quadruplex.

## Introduction

Protein dynamics is necessary for biological function as shown 40 years ago by Austin et al.^1^. It is still not known how thermally activated motions lead to functionally important conformational changes or how they might be tailored to optimize function. The question is relevant for the fundamental engineering of proteins for specific functions, understanding mechanisms of antibiotic resistance, and the development of new pharmaceutical strategies. Dihydrofolate reductase (DHFR) is an illustrative example of how protein dynamics are of both primary and applied importance. DHFR has been well studied by a variety of techniques and is one of the most important antitumor and antibacterial targets. Systematic NMR and crystallography measurements along with genetic analysis have demonstrated that on the millisecond time scale DHFR structural dynamics are species dependent, suggesting the evolutionary optimization of dynamics to the chemical environment of the organism^2^. Malaria and tuberculosis are among the diseases that are treated by antibiotics targeting DHFR in the pathogen organism. Using *Escherichia coli* DHFR (ecDHFR) it was found that the four common mutations leading to drug resistance proceed in a stepwise fashion and that the second of the four mutations, P21L, involves a residue that is not in one of the active sites, but rather is part of a dynamical loop region that allows access to the binding pocket^3^. The early development of this particular mutation shows that by altering the intramolecular dynamics of a critical protein, the bacteria attain a survival advantage.

The loop motion impacted by the ec DHFR P21L mutation can be associated with long range structural vibrations of the protein backbone that lie in the terahertz (THz) frequency range. While these vibrations had long been discussed theoretically, they have only recently been measured experimentally^4–7^. Their observation alone, however, does not provide proof of their impact on biological function. The determination of this link has been impeded by the difficulty in isolating and identifying specific motions. Typical techniques to characterize these motions have been neutron scattering, optical Kerr effect, and terahertz absorption spectroscopy^5,8,9^. These techniques typically measure broad single peaks that do not strongly vary with protein or functional state. The lack of distinct resonant features has prevented determination of specific vibrations and their impact on function. Recently anisotropic terahertz microscopy (ATM) was introduced, which uses the polarization dependence of an aligned array of proteins to isolate protein vibrations based on the direction of their dipole transition. ATM enabled the first observation of narrow band intramolecular protein vibrations^4^. The ATM spectra were found to change with inhibitor binding for the bench marking protein chicken egg white lysozyme (CEWL)^10^.

**Fig. 1:**
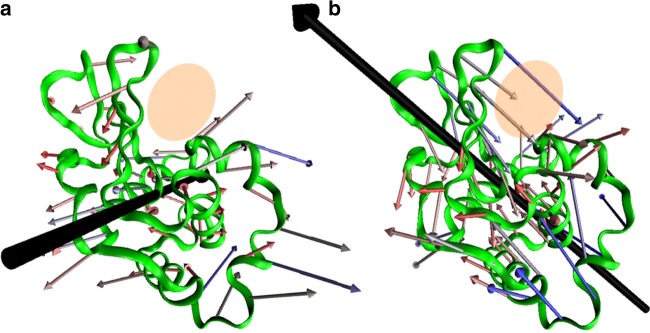
Directionality of global intramolecular vibrations. The vector diagrams of two calculated low frequency vibrations for CEWL at (**a**) 12.3 cm^−1^ (0.37 THz) and **b** 11.6 cm^−1^ (0.35 THz). The orange shaded region highlights the binding region. While the two vibrations nearly overlap in energy, their motions are dramatically different. For the 12.3 cm^−1^ case the largest amplitude motion is largely out of the page, with little motion around the binding site, whereas the 11.3 cm^−1^ vibration moves in the page with a large compressive motion into the binding region. The black arrows indicate the light polarization direction needed to excite the vibration, demonstrating that polarization dependent absorption measurements on aligned samples readily discriminates between the two vibrations

In that paper, a comparison between neutron scattering and ATM results produced the important result, confirmed by normal mode analysis, that the vibrational density of states (VDOS) is not strongly impacted by mutation or inhibitor binding, however, the directions of the vibrations are. The result was previously suggested by molecular dynamics simulations^11,12^. The importance of the impact of the directionality of vibrations is illustrated in Fig. 1, which shows the displacement vectors for two vibrations of CEWL with nearly equal energies. In spite of the similar energy of the vibrations, the backbone motion is very different. The vibration depicted in Fig. 1a has twisting motion while the vibration in Fig. 1b has compressive motion. CEWL catalyzes the hydrolysis of glycosidic bonds of alternating copolymers of N-acetyl-D-glucosamine and N-acetylmuramic acid in bacteria cell walls. The orange region in the molecules indicate the substrate binding site. The twisting motion in Fig. 1a does not strongly impact the access to the binding site whereas the hinging motion in Fig. 1b does. Measurements of the VDOS do not distinguish between the two types of motions. Thus they cannot reveal if a mutation that increases activity does so because hinging vibrations were enhanced and/or twisting motions were limited. The vibrations depicted in Fig. 1 are distinguishable by light absorption. The coupling of a vibration to light is related to the change in the molecule’s electric dipole with the vibrational excitation, the dipole transition. The exact evaluation of the dipole transition requires a full quantum mechanical treatment of a system with hundreds of atoms. For these large systems, typically the dipole transition is approximated by the semi-classical dipole derivative $\partial \vec p/\partial \vec q\sim \Delta \vec p/\Delta \vec q$, which is the change in the net molecular dipole, $\Delta \vec p$, that occurs with the vibrational atomic displacements given by the vibration vector $\Delta \vec q$. In Fig. 1, the large black arrows indicate the direction and magnitude of the dipole derivatives for the two vibrations. Only light with a component of polarization aligned along the dipole derivative can excite the vibration. Thus anisotropic absorption can provide a more detailed and specific measurement to distinguish those vibrations that impact function. However this simple picture requires either measuring a single protein, or a sample where all the proteins are aligned. To realize this macroscopic alignment, protein crystals are used. A protein crystal is a regular oriented array of proteins in an environment similar to a crowded cell with 30–70% water, most of which is mobile^13^. Because of weak intermolecular forces, protein dynamics are routinely measured using protein crystals^14–19^. As typical protein crystals are <300 μm lateral dimensions which is less than the diffraction limited spot size for the relevant frequency range, one must use a near field approach, such as used by ATM.

**Fig. 2:**
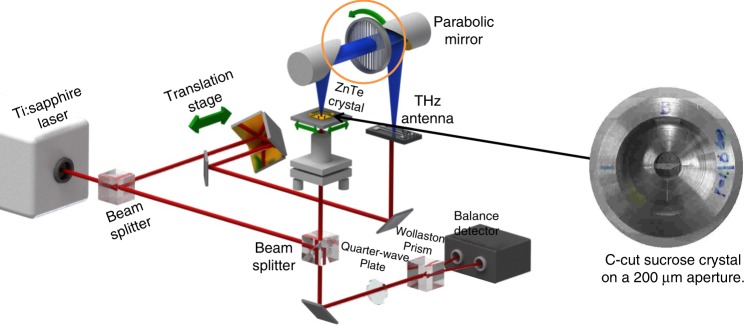
Schematic of PV-ATM optical system. Sample rotation and X-Y imaging at each rotation is eliminated by THz polarization rotation

Unfortunately ATM requires the rotation of the sample in the near field: a large technological challenge. This is in part due to the requirement that while the sample’s orientation relative to the THz polarization is changed, the orientation of the near field electro optic detection crystal must remain constant relative to the THz polarization to provide a constant response. Further, the imprecision of the sample centering on the rotation axis requires full imaging of the sample to ensure the same sample area is probed for each rotation. As a result, a complete 360-degree ATM data set requires 24 h. Here we introduce polarization varying anisotropy terahertz microscopy (PV-ATM) shown in Fig. 2, which rapidly determines anisotropic response. PV-ATM resolves closely spaced resonances that are indistinguishable in ATM. This enhanced spectral structure arises from sensitivity to sample birefringence, enabling higher specificity in fingerprinting of the global intramolecular vibrations. By removing the sample rotation requirement PV-ATM reduces a full 360-degree measurement to 4 h and provides open access to the sample for photo excitation and temperature control. We characterize and model PV-ATM using single crystal sucrose, and then demonstrate unique dynamical fingerprinting of bench marking biomolecules CEWL, DHFR, photoactive yellow protein (PYP) and RNA G-quadruplex^20^.

## Results

### Relationship of PV-ATM data to vibrational absorbance

**Fig. 3:**
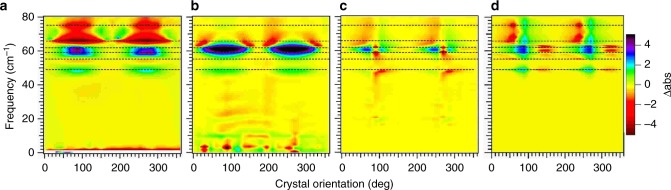
Relationship of PV-ATM data to vibrational absorbance for sucrose. The measured Δabs(*ν,θ*) using (**a**) far-field orientation dependent absorption, **b** near field anisotropic absorbance (ATM), and **c** PV-ATM. The THz radiation polarized in the *a*-*b* plane and the 0° orientation corresponds to the THz polarization parallel to the *a* axis. Also shown is simulation of the PV-ATM measurement (**d**). The simulations indicate that the increased structure in the PV-ATM spectra compared to anisotropic absorption arises from sensitivity to both the resonant anisotropic birefringence and the EO detector crystal polarization dependence

In Fig. 3 we compare surface plots of the anisotropic absorbance difference Δabs for *c* face polished sucrose single crystals measured by far field THz TDS, ATM, and PV-ATM. Sucrose readily forms molecular crystals, similar to protein crystals, except the size of the sucrose molecule forming the crystalline ordered array has a much smaller molecular weight, the crystal is stable with dehydration and the intermolecular contact forces are stronger. As sucrose single crystals can be grown sufficiently large to use standard far field techniques, the THz anisotropic absorption and birefringence are well established^21,22^. We define the anisotropic absorbance, Δabs(*ω*,*θ*), with respect to a reference polarization direction, where

1 $${\Delta {\mathrm{abs}}\left( {\omega ,\theta } \right) = {\mathrm{abs}}\left( {\omega ,\theta } \right) - {\mathrm{abs}}\left( {\omega ,\theta _{{\mathrm{ref}}}} \right) = - 2{\mathrm{ln}}\left( {E_{\mathrm{t}}\left( {\omega ,\theta } \right)/E_{\mathrm{t}}\left( {\omega ,\theta _{{\mathrm{ref}}}} \right)} \right).}$$

For all the sucrose measurements the THz light is normally incident to the *a*-*b* plane of the crystal and *θ*_ref_ = 0° is taken along the *a* axis. The far-field measurements are shown in 3 a). The positive resonances (green-blue) correspond to lattice phonons polarized along the *b*-axis and are consistent with previous measurements at 49 cm^−1^, 59 cm^−1^ and 62 cm^−1^. In spite of the close spacing, there is a clear double peak at 59 cm^−1^ and 62 cm^−1^ in the far field measurement. The negative resonances (orange-red) correspond to phonons polarized 90° from the *b* axis. The crystal *c* axis partially projects onto the *a* axis for the monoclinic crystals so these resonances include those polarized along the *a* and/or *c* axis. The resonant frequencies at 55, 66, and 75 cm^−1^ again agree with previous results. The Δabs features are periodic with the crystal rotation as expected for the oriented sample. Figure 3b shows the ATM results for a single 10 µm by 10 µm near field pixel, which do not show as much definition as the far field measurements. The resonances in Fig. 3b are coincident with those measured in the far field in Fig. 3a, including the change in polarity, however the 59 and 62 cm^−1^ resonances now appear as a single band, and only the 66 cm^−1^ *a/c* axis resonance is clearly visible. Nevertheless, the ATM spectra are directly related to the anisotropic absorption and can be readily modeled using the calculated dipole transition vectors, which for proteins can be done using normal mode analysis for example^4^. In 3c we show the PV-ATM results. There is a dramatic change in the spectrum appearance compared to the ATM and far field. The resonant peaks and valleys now appear as inflections in the polarization angular dependence. All the resonant features in the far field data are present in the PV-ATM results. In fact, it is easier to distinguish the 59 and 62 cm^−1^ resonances in PV-ATM than in the far field results. For the PV-ATM results, the vibrational dipole transition direction is given by the direction of the inflection at the resonance. While the *b* axis resonances go from negative to positive at 90 degrees, the *a/c* axis resonances go from positive to negative. To properly model the PV-ATM data, the anisotropic refractive index of the sample as well as the polarization dependence of the electro optic detection must be included in addition to the anisotropic absorbance. Using the anisotropic absorbance and birefringent refractive index previously characterized with far field measurements, our model, shown in Fig. 3d, readily reproduces the main features measured including the signs of the inflections of the Δabs angular dependence. We note the PV-ATM signal is non-zero at all polarizer orientations due to a slight ellipticity of the THz polarization at the polarizer due to mirror reflections. Evidence for this is found in Supplementary Figure 2.

### PV-ATM’s measurement of protein intramolecular vibrations

**Fig. 4:**
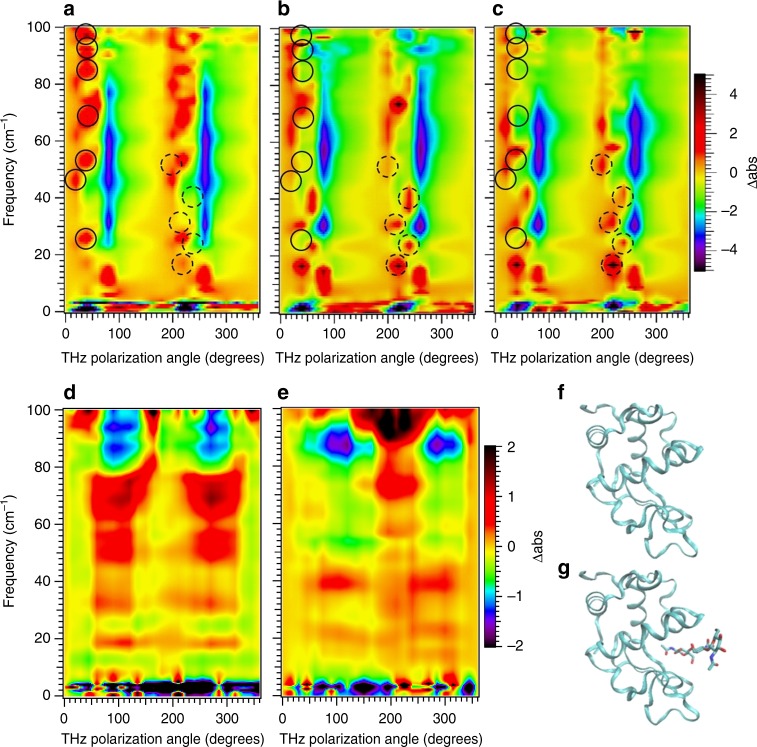
PV-ATM sensitivity to inhibitor binding. **a** CEWL; **b** CEWL-3NAG crystal 1, and **c** CEWL-3NAG crystal 2. The spectra is reproducible crystal to crystal. ATM measurements are shown for comparison (**d**) CEWL and **e** CEWL-3NAG. The simpler and faster PV-ATM technique provides spectra with increased structure over the ATM method. For the PV-ATM results solid circles indicate spectral features that are present in free CEWL and absent in inhibitor bound CEWL. Dashed circles indicate features present for inhibitor bound CEWL and absent for free CEWL. These are offset by 180° from the solid circles since all features have 180° periodicity from the regular alignment of the molecules. The large changes with inhibitor binding indicate the features arise from intramolecular vibrations and not crystal phonons. The crystal phonons are dependent on the intermolecular surface contacts and molecular mass, which change only slightly with binding as indicated by the X-ray structures for **f** free (1bwh.pdb) and for **g** inhibitor bound (1hew.pdb)

The sucrose measurements demonstrate spectroscopic reliability and sensitivity of PV-ATM. We now exploit the high spatial resolution of the near field anisotropic technique to characterize protein intramolecular vibrations. We first examine sensitivity to inhibitor binding using CEWL and the inhibitor tri acetyl glucosamine (3NAG)^10^. Previously it was found that inhibitor binding only slightly alters the broad featureless VDOS and isotropic absorption, while anisotropic absorption readily detects the reorientation of intramolecular vibrations. Figure 4 shows the PV-ATM results for CEWL with the THz light incident on the (110) face of tetragonal crystals, identical to the sample configuration used in the previous ATM measurements. Figure 4a shows the free CEWL PV-ATM spectrum and Fig. 4b, c show 3NAG bound CEWL spectra for two different crystals. The PV-ATM spectrum has more distinct features than the previous ATM measurements^4,10^. Most likely the broad ATM spectral resonances break up into distinct features in PV-ATM due to the rapid refractive index variation accompanying closely spaced resonances, as is seen for the 59 and 62 cm^−1^ resonances of sucrose in Fig. 3. Comparing 4a to 4b, and 4c we see that the protein dynamics are strongly altered by the inhibitor binding as was reported previously using ATM, however PV-ATM shows considerably more spectral and angular structure. PV-ATM Resonances removed with binding are indicated by solid line circles, whereas new resonances arising with binding by dashed line circles. To ensure clarity between the two different sets of resonances, we use the 180° periodicity and indicate the free CEWL resonances at or near 40° and the inhibitor bound CEWL resonances at or near 220°. A comparison between Fig. 4b, c shows good crystal to crystal spectral reproducibility. Some of the differences between the two correspond to features present in the free CEWL, suggesting that they may arise from differences in inhibitor binding. For example the resonance at 72 cm^−1^ // 220° is not seen in either the free CEWL or the second 3NAG-CEWL crystal. It is possible that this resonance is due to a free excess 3NAG in the first 3NAG-CEWL crystal. While we expect the underdamped intramolecular vibrations of free 3NAG at higher frequencies^23–25^, interstitial 3NAG bonding within the protein lattice may lead to this optically active vibration. The PV-ATM method provides a quick and sensitive determination of the presence of inhibitor binding.

**Fig. 5:**
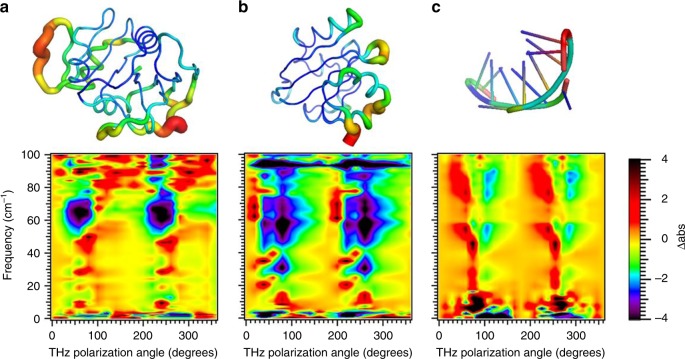
Unique dynamical fingerprints for different biomacromolecules. PV-ATM spectra for **a** *E. coli* DHFR (1dra.pdb); **b** photoactive yellow protein (2phy.pdb) and **c** RNA G-quadruplex (4xk0.pdb) rendered with Pymol B-factor putty. B-factor varies from high (red) to low (blue). While the vibrational density of states, that is the vibrational energy spectrum, for the three systems are highly similar, the actual motion for a given vibrational energy is structure specific leading to large differences in the anisotropic response

The uniqueness of spectra for different biomolecules is illustrated in Fig. 5 which shows the PV-ATM spectra for photoactive yellow protein (PYP), DHFR and RNA G-quadruplex. The PV-ATM spectra for the three systems are readily distinguishable. Of particular note is the fingerprint of the quadruplex. We found the chevron structure reproducibly for several RNA G-quadruplexes with different crystal structures, but not for any protein. It is possible that this is signature of the low frequency optical response of the quadruplex structure.

## Discussion

The sucrose data in Fig. 3 shows a striking increase in structure for the PV-ATM data over the ATM, with the absorbance resonances at 59 and 62 cm^−1^ easily distinguished (see Supplementary Fig. 1). As mentioned earlier, modeling of the PV ATM signal shown in Fig. 3d reproduces these sharp features only when the frequency dependent sample birefringence is included. This result reveals the added structure in the case of sucrose is coming from the polarization rotation of the THz. The near field EO detection is strongly dependent on the relative THz and NIR probe polarizations. We account for this known variation by using an empty aperture reference measurement. For the THz polarizer at 90° the relative polarizations are such that the signal is at minimum. Thus any rotation of the THz polarization by the sample will result in an enhanced EO response. The birefringence that necessarily accompanies the anisotropic absorption lines of the sucrose provides this frequency dependent polarization rotation for the sucrose. For the biomacromolecules the additional structure over that of the ATM measurements likely arises in part from the accompanying birefringence for different resonances as well. However we cannot ignore the possibility that the increase in the EO signal could also come about from circular dichroism resonances. It has been speculated that circular dichroism can occur at these frequencies for biomolecules. The ATM technique would not have been sensitive to this effect as the relative polarization of the THz and NIR probe were held constant for the entire measurement. We are currently testing for this possibility.

Sucrose, protein and RNA crystals all consist of a regular array of molecules forming a lattice, however the origin of the THz resonances for the sucrose crystals is distinctly different from the resonances for the proteins. The sucrose anisotropic resonances seen in Fig. 3 arise from intermolecular vibrations, phonons, between sucrose molecules within the lattice. These vibrations can be visualized as net displacements of the entire molecule at a given crystal lattice site relative to the neighboring molecules. For the proteins on the other hand, the resonances arise from intra molecular vibrations: vibrations within each molecule rather than between molecules. This is apparent from the frequencies of the PV-ATM resonances for all the proteins measured. We can approximate the lattice vibrational frequency as $\sqrt {k/m}$, where *m* is the mass of the molecule and *k* is the effective force constant of the intermolecular bonds with neighboring molecules. The protein mass is ~1000 times that of sucrose. While at the same time the intermolecular bond strengths for protein crystals are less than half those of sucrose crystals^26^. This weak intermolecular protein interactions are evident in the extreme fragility of protein crystals. The two factors together lead to protein crystal lattice phonon frequencies of less than 3 cm^−1^ ^27,28^, a factor of 10 smaller than the observed resonances. On the other hand the PV-ATM protein resonant frequencies are consistent with normal mode analysis estimates of intramolecular vibrations^29^.

The CEWL binding measurements shown in Fig. 4 are an additional confirmation that the observed resonances are intramolecular vibrations. In Table 1 we show the frequency of the Δabs peaks observed for CEWL that are not present in the CEWL-3NAG, as well as those observed for CEWL-3NAG not present for CEWL. There are obvious large changes in the spectra with inhibitor binding. For example the peak at 26 cm^−1^ //40° is not present in the 3NAG bound sample, but instead three peaks at 16 cm^−1^ //40°, 24 cm^−1^ //60° and 31 cm^−1^ //40° are present. The 26 cm^−1^ resonance must either shift in frequency, shift in direction or no longer exist in the bound state. In all three cases we can rule out this resonance arises from the crystal lattice phonons. The maximum frequency red shift for lattice phonons is ≤2 % with inhibitor binding due to mass loading. In addition, there would be no reorientation of the polarization of the phonon since all the CEWL and CEWL-3NAG crystals measured belong to the same tetragonal space group (P4_3_2_1_2), and all measurements are for the same crystal face and reference polarization direction. The large frequency shifts and/or orientation changes must then arise from changes in the intramolecular vibrations.

**Table 1:** PV-ATM Δabs peaks from CEWL and CEWL-3NAG crystal measurements

CEWL				CEWL-3NAG			
*ν*	Δν	*θ*	Δabs	*ν*	Δν	*θ*	Δabs
26	3	40°	2.78	16	4	40°	4.77/5.39
46	4	20°	1.50	24	2	60°	1.07/1.08
53	4	40°	2.36	31	3	40°	1.61/1.28
68	4	40°	2.54	41	6	60°	1.65/1.22
85	6	40°	2.84	51	3	20°	0.42/1.27
92	3	40°	3.44				
97	3	40°	3.63				

The table shows the Δabs peak frequencies, *ν* (cm^−1^), linewidths, Δ*ν* (cm^−1^), angles, *θ*, and Δabs that arise in the CEWL crystal spectrum only (left side) and the CEWL-3NAG spectra only (right side). The intensities for CEWL (CEWL-3NAG) listed for Fig. 3a (Fig. 3b, c)

We now turn to the possible interpretation of the resonances in Table 1. Focusing first on the <60 cm^−1^ resonances, the CEWLresonances at 26 cm^−1^, 46 cm^−1^, 53 cm^−1^ are replaced by 16 cm^−1^, 24 cm^−1^, 31 cm^−1^, 41 cm^−1^, and 51 cm^−1^ for CEWL-3NAG. A straight forward interpretation of the observed changes is that resonances that were previously associated with displacements into the substrate site are eliminated by the binding and new distinct vibrations associated with the changes in the internal couplings emerge. Referring back to Fig. 1, the vibration depicted in Fig. 1a would be unaffected by the occupancy of the substrate site, whereas the vibration in Fig. 1b has displacements that would be eliminated by binding. Then resonances at 26 cm^−1^, 46 cm^−1^, 53 cm^−1^ may all directly influence substrate access. However it is possible that increased internal coupling with substrate binding results in the 26 cm^−1^ and 46 cm^−1^ resonances blue shifting to the similarly oriented 3NAG bound 31 cm^−1^ and 51 cm^−1^ resonances. Assuming this is the case, this then isolates the 53 cm^−1^ resonance as a candidate vibration to modulate substrate access. We now examine the >60 cm^−1^ resonances, which appear to be absent or reoriented and decreased in intensity for CEWL-3NAG. The dramatic change in this frequency range is similar to the ATM results. It is important to reemphasize that the molecular orientation is exactly the same for the different measurements, thus the reorientation of a resonance either entirely or partially out of the plane corresponds to substantial impact on the collective atomic displacements. All resonances in this range are good candidates for examining the influence of these motions on biological function. Tests of this relevance could take the form of strong excitation at the candidate resonance frequency and monitoring ligand binding rates and/or the net catalytic rate. Challenges to this test are catalytic changes due to solvent heating with intense excitation and the relatively slow CEWL catalytic rate.

The unique PV-ATM spectra for CEWL, PYP, DHFR, and the RNA G-quadruplex are reminiscent of the small molecule optical fingerprinting in the mid infrared (MIR) range 500–1800 cm^−1^. Specific bonds and/or neighboring bonds result in vibrational energies, which can be optically measured. The resonant absorption lines provide a signature of the specific chemical compound. The spectra can be complex, and the assignment of lines to specific motions is challenging. However, because the spectra are compound specific, chemical identification is achieved via established databases, where the MIR spectra have been catalogued^30,31^. Thus MIR spectroscopy has become a workhorse for environmental monitoring and other chemical sensing applications^32^.

The number of absorbance lines in the MIR quickly increases with the size of the system. For proteins this is particularly true since the polypeptide chain has repeating peptide bonds as well as multiple instances of individual residues. As a result, MIR spectra of proteins have broad absorption bands that look nearly identical for different proteins. Protein MIR measurements suffer from the protein amide I band overlapping a liquid water strong vibrational line at ~1640 cm^−1^, similar to the background issues for THz measurements. The water overlap in the MIR is addressed by using D_2_O as the solvent, with a strongly red shifted vibration to ~1210 cm^−1^. While MIR cannot be used for protein fingerprinting, it is used to monitor changes in a protein’s secondary structure during function or after a perturbation^33–35^.

Significant strides in the precision of IR determination of secondary structure content has been made using increasingly more sophisticated techniques such as 2D IR on deuterated samples^36^. As different proteins can have essentially identical secondary structure content, these measurements cannot be used as fingerprints of a specific protein. The vibrations in the far infrared (FIR), 2–400 cm^−1^, correspond to global motions which involve relative motions between domains and are dictated by the 3D structural arrangement of the alpha helices, beta sheets and unstructured regions. The vibrational frequencies and optical-dipole couplings (magnitude and direction) are dependent on the intramolecular interactions throughout the macromolecule, and thus are unique to each protein. While the long range vibrational spectra of proteins were first calculated many years ago^37^, they have been difficult to measure due in part to the overlap of the vibrational energies with bulk water absorption. In contrast to MIR, solvent replacement is not effective for FIR measurements as there is very little change in the strong broadband absorbance between H_2_O and D_2_O^38^.

While careful and precise isotropic absorbance measurements have detected changes in the broad absorption peaks with substrate/inhibitor binding for a variety of proteins^39,40^, the polarization dependent THz absorption methods used here remove the isotropic water absorption and increase detection selectivity, isolating resonant bands based on the direction of the transition dipoles. It is notable that the near field measurements reflect the net absorbance for a large population of biomolecules which are distributed over a rugged energy landscape. The fact that well-defined spectral resonances are observed for the ensemble suggests that there are preferential motions dictated by the overall architecture of the protein’s energy landscape. These preferential motions can lead to biased sampling of specific regions of configurational space which enable efficient conformational transitions^41^. The changes in the PV-ATM spectra with inhibitor binding shown in Fig. 3 indicate a reorientation of the vibrational motions resulting in the backbone exploring a different region of configurational space. For example, Fig. 3a, b indicate that the motions with net displacements in a direction 40° from the [001] direction decrease with inhibitor binding, whereas motions at 60° from the [001] direction increase. Ideally specific motions could be identified through comparisons with calculations. While such spectral analysis may be possible for the ATM method, where the resonant bands for proteins have already been qualitatively reproduced using normal mode analysis, the PV-ATM simulations for sucrose indicate that it is essential to include the anisotropic refractive index to accurately calculate the spectrum. Current protein modeling cannot readily predict this level of dielectric response, making precise assignment of PV-ATM features out of reach at this time. Nevertheless, the simplicity of the technique along with high spectral definition makes the monitoring of biomacromolecule global structural dynamics easily accessible. The CEWL measurements for example illustrate the application of THz PV-ATM as a critical diagnostic to determine if complex formation is present in a biomolecular crystal before submission for high resolution crystallography.

We note that in addition to the spectral fingerprinting we show here, because PV-ATM is based on THz pulsed sources, there is an opportunity to time resolve changes in protein internal dynamics. For example PYP has been a test case for many time resolved dynamic characterization methods^42–44^, most notably time-resolved X-ray crystallography^14,17–19,45–47^.

Mutation studies^48,49^, NMR dynamical mappings^2,50,51^, and numerous computational studies^52,53^ have indicated that distant structural regions in proteins are linked. Global vibrations of the backbone are thought to provide a simple mechanism for that linkage, however accessible measurements of these motions have been lacking. PV-ATM is a simple fast measurement that can immediately determine if and how global vibrations impact biological function, and how these motions can be steered by mutation and binding within the dynamical network. The anisotropic absorbance overcomes the challenges from the large vibrational density of states and solvent background, enabling isolation of specific structural vibration bands. This is a considerable improvement over the featureless VDOS and isotropic absorbance spectra. While PV-ATM is far more accessible than ATM, it is still reliant on crystals to provide the uniform alignment of the macromolecules. Ideally one could realize molecular orientation control within solutions and perform these measurements in the far field. The rapid advancements in microfluidics may make this possible through either electrostatic or laminar flow alignment^54^.

## Methods

### Sample methods

Sucrose powder is supplied by Sigma Aldrich. Sucrose was first dissolved in pure deionized water to saturation at 23 °C. Then small sucrose seeds were grown by drying the saturated solution. Seeds were further hung in a beaker filled with clean saturated solution for several days at room temperature. The grown crystals were finally polished by hand along the *c* face to have a large area and are sufficiently thin so that the absorbance is within the dynamic range of the terahertz system^22^.

The sucrose, CEWL, CEWL-3NAG, and PYP samples measured by ATM and PV-ATM are mounted on a 200 µm thick steel disk with a center drilled 200 µm hole. The hDHFR and ecDHFR samples are mounted on a 100 µm thick *c* face sapphire disk with a ~6 µm thick aluminum coating on the top surface and a 300 µm aperture in the center of the metal coating. Samples are placed directly on top of the aperture. We have found good reproducibility between different crystals produced under similar growth conditions. We also have found some crystal aging effects, and believe this should be further investigated, as well as pH and salt concentration effects^55–57^.

Of particular importance is the maintenance of sample hydration for the protein crystals, which are typically greater than 0.5 g water/g protein. First the protein crystal samples are covered with a small drop of paraffin oil to maintain crystal hydration throughout the experiment, as is common practice in protein crystallography. The paraffin oil is transparent and isotropic at THz frequencies, as discussed in earlier papers. In addition, the sample sits in a hydration-controlled chamber for the measurement. The details of the construction of this chamber are in the ATM section below. Inspection of all samples before and after the measurements found no cracking associated with drying. The maintenance of hydration is critical for two main reasons. First, as the crystals are ~50% water, removal of this water will decrease crystal order and protein alignment. Secondly, if the crystals are sufficiently dehydrated, the biological water that is necessary for the native 3D structure and dynamics will be impacted. In vivo structure requires a minimal hydration level, typically 0.3 g water/g protein. For all the biomacromolecules studied, the hydration level is higher than 0.5 g water/g protein. The CEWL and CEWL-3NAG crystals are aligned on the aperture such that the THz is incident on the (110) crystal face and the 0° THz polarization is aligned along the [001] axis of the protein crystal, as determined by the crystal facets. The PYP hexagonal crystal is aligned such that the 0° THz polarization is aligned along the *c* axis of the crystal. The ecDHFR crystal facets are not as easily identifiable and so the 0° THz polarization direction relative to the crystal alignment is unknown.

The free CEWL crystals are grown at room temperature with the sitting drop method^4^. The protein was purchased from Sigma-Aldrich. It was prepared at 60 mg ml^−1^ in 0.1 M NaAc pH 5.2 buffer without further purification. The precipitant is 10% NaCl in the same buffer. The sitting drop contained 10 µl of precipitant and 10 µl of protein solution with 500 µl of precipitant in the reservoir. CEWL crystals with 3NAG inhibitor are grown in the same manner, with the exception of an equal concentration of 3NAG as the protein added to the protein solution. The CEWL and CEWL-3NAG crystals have a tetragonal structure, space group P4_3_2_1_2.

*E. coli* DHFR (ecDHFR) is obtained from Sigma-Aldrich, and crystallized with a protein concentration of 9.4 mg ml^−1^ and 4 °C growth temperature using hanging drop method^58^. The recipe of the buffer includes 300 mM manganese (II) chloride, 14% polyethylene glycol 6000, and 20 mM imidazole. The buffer was adjusted to pH 7.0. The ecDHFR had space group P6_1_. Characterization measurements after crystallization show the ecDHFR is MTX bound with NADPH not present.

PYP was directly purified from E. halophila strain BN9626, originally isolated by Meyer et al.^59^. First a microseed solution was made by grinding large, grossly twinned crystals in 3.2 M ammonium sulphate and 20 mM sodium phosphate, pH 7.0. Then the hanging drops of 10 µl protein solution (15 mg ml^−1^ PYP in 2.5 M ammonium sulphate and 20 mM sodium phosphate, pH 7.0) on siliconized coverslips were injected with 0.5 µL of microseed solution, over 500 µl reservoirs of 2.5 M ammonium sulphate^60^. The resulting crystals are needle-like with a hexagonal structure, space group P6_3_. The long axis of the needle corresponds to the *c* axis and the THz light is incident on the (120) face.

RNA G-quadruplex crystallization conditions are using hanging drop method^20^. The recipe for the reservoir is 40 mM potassium cacodylate (pH 6.0), 35% 2-methyl-2,4-pentanediol, 5 mM spermine hydrochloride, 80 mM potassium chloride, 0.5 mM DHX36 fragment, and 20 mM barium chloride. The mixture of hanging drop contains 2 mM solution of the RNA, 10 mM HEPES potassium hydroxide (pH 7.5) and 100 mM potassium chloride, in a 1:1 ratio with the reservoir solution. The tetragonal crystals (space group P42_1_2) were shaped as square plates, and the broad face of the crystals is perpendicular to the *c* axis. THz light is incident on the (001) face, with the reference direction along (100) or the equivalent (010).

### THz TDS

TDS measurements were performed using THz time domain spectroscopy^61,62^ using photoconductive gallium arsenide antenna generation^63^ and zinc telluride electro optic detection. The 800 nm laser generated from a titanium doped sapphire laser (Tsunami, 100 femtosecond, Spectra-physics) is split into the pump and probe beams by a polarizing beamsplitter. The pump beam is focused on the antenna to generate THz emission, and then collected by a hyper-hemispherical silicon lens. The probe pulse propagates via a mechanical delay line for changing the delay time between pump and probe arms. Transmission measurements of single crystal sucrose are performed in 15° rotations from 0–360° where 0° denotes the THz polarization aligned along the *a* axis. A resolution of 0.68 cm^−1^ was used for all measurements, THz TDS, ATM and PV-ATM. The resolution was chosen based on previous calculations of protein anisotropic bands^10^ and the measured linewidths for single crystal molecular crystals such as sucrose, fructose and oxalic acid^22,64^. These crystals have strong lattice phonons in the THz range and typical linewidths of 2 cm^−1^ or greater at room temperature.

### ATM

Anisotropy Terahertz Microscopy (ATM)^4^ is a near-field measurement, where the sample is in nearly direct contact with an electro-optic detection crystal. This enables measurements of samples, such as protein crystals, which are significantly smaller than the terahertz diffraction limited spot size. The sample is centered on a sample plate with a THz transparent aperture. The sample plate is placed directly on the electro optic detection crystal such that the aperture is over the near infrared (NIR) probe incident on the electro optic crystal from the bottom surface. The electro optic crystal also acts as the bottom of an environmental chamber for the sample. The hydration within the chamber is controlled by airflow from a Licor Dewpoint Generator. For the sucrose measurements, the hydration lid is in place, but no hydrated air is flowing. The THz light is incident on the sample from the top and transmission through the sample and aperture are probed with NIR which is incident from below the electro optic crystal. The NIR is reflected off of the top surface of the electro optic crystal, co propagates with the transmitted THz and is then guided to a balance detector, similar to a standard THz TDS setup. The sample is rotated by 15° increments using an automated rotation stage. For each rotation, the sample plate is lifted by automated actuators above the EO crystal, the sample plate is rotated and then lowered back onto the EO crystal. This allows for a controlled rotation measurement without breaking the dry nitrogen gas purge surrounding the THz system. This improves measurement times and resolution of previous ATM measurements^4^. At each orientation, a raster scan is performed to determine the position of the aperture center and three high resolution measurements are performed to obtain the transmitted THz electric field, at the center position and ± 30 µm off-center. The three waveforms are averaged for each orientation before performing a Fourier transform to determine the power spectrum. The Δabs is defined as difference in the sample absorption at a specific orientation relative to the reference orientation, as in the relation below

2 $$\Delta {\mathrm{abs}}\left( {\omega ,\theta } \right) = - 2{\mathrm{ln}}\left[ {\frac{{\left| {E_{\mathrm{t}}\left( {\omega ,\theta } \right)} \right|}}{{\left| {E_{\mathrm{t}}\left( {\omega ,\theta _{{\mathrm{ref}}}} \right)} \right|}}} \right] = - 2{\mathrm{ln}}\frac{{F\left( \omega \right)\left| {E_i\left( \omega \right)} \right|e^{ - \alpha \left( {\omega ,\theta } \right)d/2}}}{{F\left( \omega \right)\left| {E_i\left( \omega \right)} \right|e^{ - \alpha \left( {\omega ,\theta _{{\mathrm{ref}}}} \right)d/2}}} \\ = \left[ {\alpha \left( {\omega ,\theta } \right) - \alpha \left( {\omega ,\theta _{{\mathrm{ref}}}} \right)} \right]d$$

Where Δabs is the relative absorption, *E*_t_ is the transmitted THz electric field, *ω* is the frequency, *θ* is the orientation, *θ*_ref_ is the reference orientation 0°, *d* is the sample thickness and *α*(*ω*,*θ*) is the absorption coefficient. This self-referencing removes any narrow band atmospheric water absorption from the sample hydration chamber.

### PV-ATM

In order to do anisotropic measurements, one must rotate the sample relative to the THz polarization. This rotation of the sample is challenging due to its proximity to the electro optic detection crystal. Each time the sample is rotated relative to the electro optic crystal, the displacement of the sample center must be determined. In addition, one must account for the pixel-to-pixel variation in the electro optic crystal. As a first order method to speed up and simplify the measurement process, we rotate the terahertz polarization incident on the sample by rotating a wire grid polarizer mounted on an automated stage.

The polarization-varying ATM (PV-ATM) experimental setup is similar to the described ATM setup with the addition of a wire grid polarizer on an automated rotation stage which is located on the THz arm of the near field microscope. A schematic of the PV-ATM system can be found in Fig. 1. The wire grid polarizer has an electric field extinction ratio, $t_\parallel /t_ \bot$, of ~20:1 and a power extinction ratio of 400:1. Time domain THz transmission measurements to obtain the THz amplitudes and phases are taken in the near field taken at the center of the aperture every 20° of rotation for a sucrose measurement, and every 15° of rotation for a protein measurement. If the input THz light is linearly polarized, the transmitted THz changes by the factor cos(*θ*) where *θ* is the angle of the polarizer relative to the initial polarization direction. The electro optic detection crystal is also polarization sensitive, so rotating the THz polarization also modulates the detection sensitivity^65^. To account for this, sample measurements at each THz polarization are referenced to an empty hole. A full PV-ATM measurement consists of first measuring an empty aperture for a full rotation of the polarizer followed by a full polarization rotation measurement of the sample. Sample measurements are referenced to the empty aperture and the relative absorption is determined using Eq. 1. All measurements are performed at room temperature. Hydration of the sample is maintained by a Licor Dewpoint Generator.

PV-ATM sucrose measurements are performed on the same sample as in the ATM measurements. The sample is not removed from the THz system between the two experiments, so the 0° orientation is the same for each experiment. Hydration control for the protein samples is achieved in the same way as in the ATM system, with both paraffin oil coating the sample and a hydration-controlled cell using hydrated air flow. For the sucrose measurements, the hydration lid is in place, but no hydrated air is flowing. We note the strong negative relative absorption feature at 80° THz polarization angle, present in the CEWL and CEWL-3NAG crystal results, but not present in the other spectra. This is due to a net rotation of the probe NIR polarization relative to the electro optic crystal axes, which was removed for subsequent measurements.

### Simulation and modeling methods

The sucrose PV-ATM simulations are performed by modeling the THz dielectric response of a *c* cut sucrose crystal, and calculating the expected THz PV-ATM response. The dielectric response of the sample is modeled using the far-field THz absorption of the *c* cut sucrose crystal, shown in Fig. 2a. We fit the 0° and 90° absorption spectra with multiple Lorentzian peaks and a baseline function to obtain the peak amplitudes, frequencies, and peak widths (*A*_*n*_, *ω*_*n*_, *y*_*n*_, respectively). The fit parameters are then entered into the following permittivity equation:

3 $$\varepsilon \left( \omega \right) = \varepsilon _{{\mathrm{DC}}} + \mathop {\sum }\limits_{n = 0} \frac{{1/2A_n}}{{\omega _n^2 - \omega ^2 - i.\omega .\gamma _n}},$$

where *ε*_DC_ is the DC dielectric constant, *ω* is the frequency, and *n* is the peak number. We obtain two permittivity expressions *ε*_*o*_(*ω*) and *ε*_*e*_(*ω*) by using the 0° and 90° fit parameters, respectively. The DC dielectric constant is set for each permittivity equation such that the index of refraction, ${\mathrm{Re}}\left( {\sqrt {\varepsilon \left( \omega \right)} } \right)$, at 0 cm^−1^ is 1.8.

The expected THz signal is determined by first using Jones matrix calculus to obtain the transmitted THz magnitude and rotation angle through the polarizer and sucrose sample. The resulting transmitted electric field vector polarization relative to the EO crystal is then used to calculate the detected THz. As with the measurements, simulations are performed for both the sample and the reference. The initial THz polarization is set to the *x* direction of the lab frame. The electric field vector is then rotated into the reference frame of the polarizer, which lies at an angle *θ* relative to the *x* direction, is transmitted through the polarizer, and then is rotated back to the lab frame. The transmitted electric field through the sample and polarizer is calculated similar to the reference with the addition of the sample frequency dependent anisotropic dielectric functions. For the anisotropic response of the *c* cut sucrose sample we label the *a* axis as the ordinary axis and the *b* axis as the extraordinary axis. The initial orientation of the sample is defined by the rotation of the *a* axis relative to the lab frame and is labeled *β*. The electric field vector transmitted through the polarizer is rotated into the sample frame, with rotation angle *β*, is transmitted through the sample, and then is rotated back to the lab frame. The transmission matrix for the sample is diagonal with $t_{xx} = e^{i\sqrt {\varepsilon _o\left( \omega \right)} 2\pi \omega d}$ and $t_{yy} = e^{i\sqrt {\varepsilon _e\left( \omega \right)} 2\pi \omega d}$, where *d* is the sample thickness, and *d* = 0.0275 cm for the sample measured. These procedures are used to obtain the transmitted THz magnitude and angle of rotation for both sample and reference.

Due to the polarization sensitivity of the EO response, the detected THz signal from the experiment modeled using the modified Planken expressions^65^:

4 $${\begin{array}{l}{\mathrm{\Delta }}I_{{\mathrm{ref}}}\left( {\omega ,\theta } \right) = \left| {E_{{\mathrm{ref}}}\left( {\omega ,\theta } \right)} \right|\left[ {{\mathrm{cos}}\left( {\phi _{{\mathrm{ref}}}\left( {\omega ,\theta } \right) + \phi _0} \right){\mathrm{sin}}\left( {2\varphi } \right) + 2{\mathrm{sin}}\left( {\phi _{{\mathrm{ref}}}\left( {\omega ,\theta } \right) + \phi _0} \right){\mathrm{cos}}\left( {2\varphi } \right)} \right]\,{\mathrm{and}}\\ {\mathrm{\Delta }}I_{{\mathrm{sample}}}\left( {\omega ,\theta } \right) = \left| {E_{{\mathrm{sample}}}\left( {\omega ,\theta } \right)} \right|\left[ {{\mathrm{cos}}\left( {\phi _{{\mathrm{sample}}}\left( {\omega ,\theta } \right) + \phi _0} \right){\mathrm{sin}}\left( {2\varphi } \right) + 2{\mathrm{sin}}\left( {\phi _{{\mathrm{sample}}}\left( {\omega ,\theta } \right) + \phi _0} \right){\mathrm{cos}}\left( {2\varphi } \right)} \right],\end{array}}$$

where *ϕ* and *φ* are the THz and NIR polarization angles relative to the [001] axis of a (110) cut EO crystal, and *ϕ*_0_ is the initial THz polarization angle. The THz and NIR are incident on the [110] EO plane, perpendicular to the surface. The initial THz polarization angle, *ϕ*_0_, and the NIR angle, *φ*, are determined by fitting Δ*I*_ref_(33 cm^−1^,*θ*) vs *θ* and until there is good agreement between the data and simulation. In the case of the initial PV-ATM sucrose simulations *ϕ*_0_ = −5.12° and *φ* = 48.45° give the best agreement (see Supplementary Fig. 2).

The change in THz signal due to the polarizer is normalized by referencing each polarizer rotation angle through

5 $$\left| {T_{{\mathrm{sample}}}\left( {\omega ,\theta } \right)} \right| = \frac{{{\mathrm{\Delta }}I_{{\mathrm{sample}}}\left( {\omega ,\theta } \right)}}{{{\mathrm{\Delta }}I_{{\mathrm{ref}}}\left( {\omega ,\theta } \right)}},$$

and the relative absorption is determined through

6 $${\mathrm{\Delta }}{\mathrm{abs}}\left( {\omega ,\theta } \right) = - 2{\mathrm{ln}}\left( {\frac{{\left| {T_{{\mathrm{sample}}}\left( {\omega ,\theta } \right)} \right|}}{{\left| {T_{{\mathrm{sample}}}\left( {\omega ,0^{\mathrm{o}}} \right)} \right|}}} \right).$$

This is calculated for *ω* = 0,0.1,…,200 and *θ* = 0,15,…,360 and the spectra is plotted as a surface plot as shown in the Fig. 3d. The orientation of the sucrose crystal relative to the THz polarization is unknown, so the sample orientation angle, *β*, is adjusted until the obtained spectra is in good agreement with the measurement. Here an orientation angle of *β* = −75° is used as is gives the best agreement with the experimental data.

### Reporting summary

Further information on experimental design is available in the Nature Research Reporting Summary linked to this article.

## Supplementary information

Reporting Summary

Supplementary Information

## Data Availability

Data supporting the findings of this manuscript are available from the corresponding author upon reasonable request. A reporting summary for this Article is available as a Supplementary Information file.
