# Supplementary material for: Protein and RNA dynamical fingerprinting
Source: Nat Commun. 2019 Mar 4;10:1026. doi: 10.1038/s41467-019-08926-3 (PMC6399446; doi:10.1038/s41467-019-08926-3)
Supplement: Supplementary file 1 — Reporting Summary [file 41467_2019_8926_MOESM1_ESM.pdf]

<u>Supplementary Information</u>

Protein and RNA Dynamical Fingerprinting

K. A. Niessen et al.

Supplementary Information

Protein and RNA Dynamical Fingerprinting

Katherine A. Niessen [1], Mengyang Xu [1], Deepu K. George[1], Michael C. Chen[2], Adrian R. Ferré-D'Amaré[2], Edward H. Snell [3], Vivian Cody[3], James Pace[3], Marius Schmidt[4], and Andrea Markelz [1,3]

[1] Department of Physics, University at Buffalo, SUNY, Buffalo, New York, USA
[2]National Heart, Lung and Blood Institute, Bethesda, Maryland, USA
[3]Hauptman-Woodward Medical Research Institute & Department of Structural Biology, University at Buffalo, SUNY, Buffalo, New York, USA
[4]Department of Physics, University of Wisconsin, Milwaukee, Wisconsin, USA

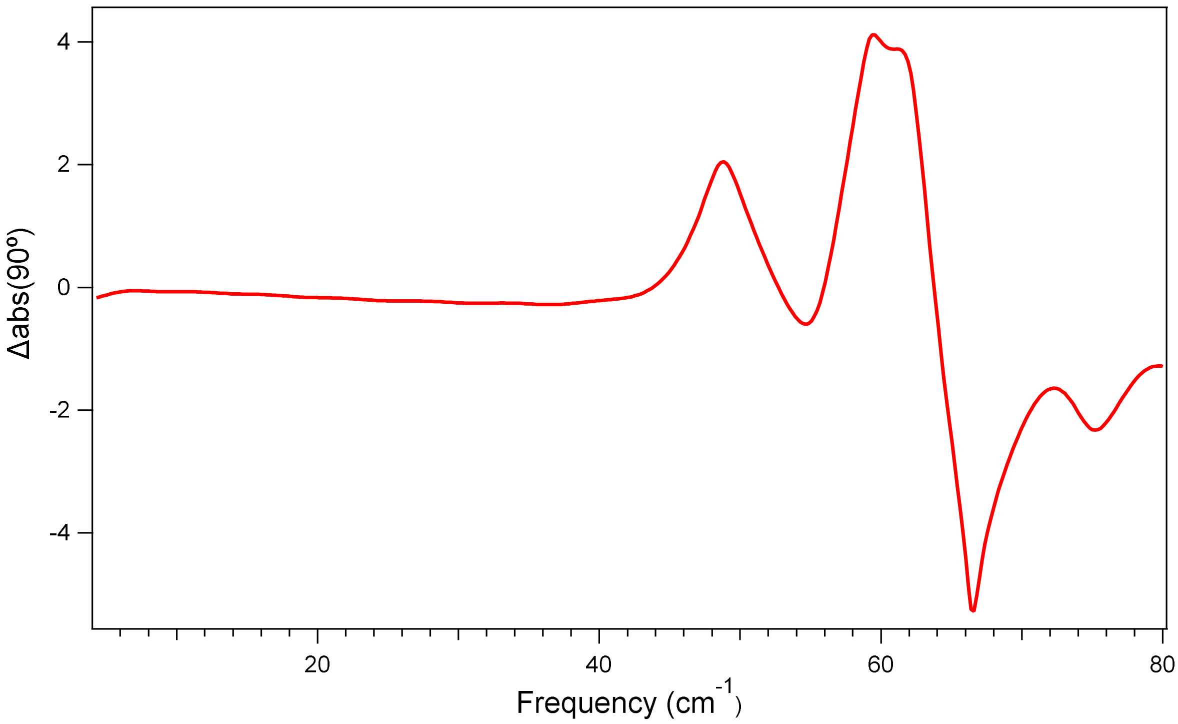

Supplementary Figure 1. Far field Δabs of sucrose extracted from Fig. 2 of main text at 90°.The far field measurement shows distinct resonances at 59 cm^{-1} and 62 cm^{-1}, which are not resolved by near field ATM. The higher spectral resolution of near field PV ATM resolves these

PV ATM simulation details

The calculations are performed by first using the Jones Matrix equations to obtain the transmitted THz then using the expected EO response to determine the detected THz. As with the measurements, simulations are done for both the sample and the reference and the magnitude of

transmission through the sample is determined by |T(ω,θ)|. The change in absorption relative to the a 0° polarizer rotation is $\Delta abs(\omega,\theta) = -2\ln\left(\frac{|T(\omega,\theta)|}{|T(\omega,0°)|}\right)$.

The dielectric response of the sample is modeled using the far-field THz absorption of the c-cut sucrose crystal. We fit the 0° and 90° absorption spectra with multiple Lorentizian peaks and a

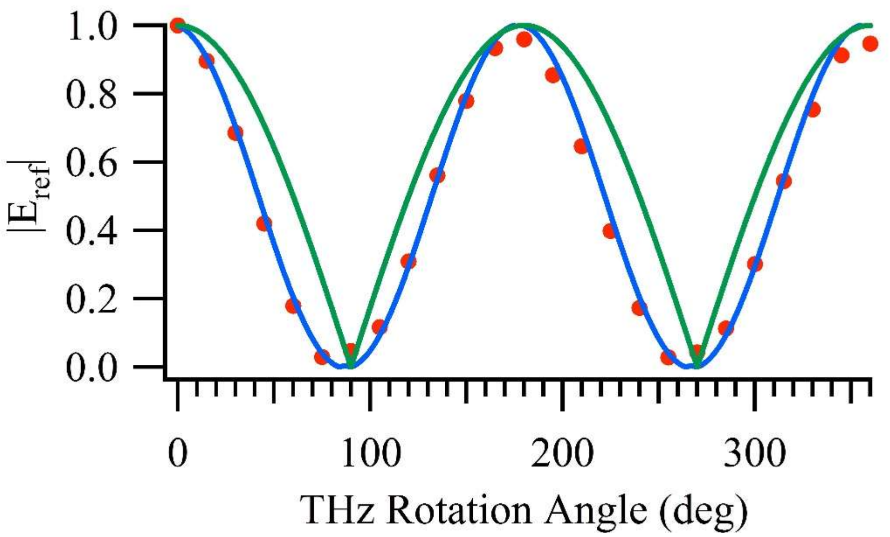

Supplementary Figure 2. Measured PV-CATM reference signal (red dots), along with |cos(q)| fit (green line) and calculated signal including THz polarization dependence of electro optic response (blue line).

baseline function, to obtain the peak amplitudes, frequencies, and widths (A_n , ω_n , and γ_n, respectively). The fit parameters are then entered into the following permittivity equation: $\varepsilon(\omega) = \varepsilon_{DC} + \sum_{n=0} \frac{1/2\,A_n}{\omega_n^{\,2} - \omega^2 - i\cdot\omega\cdot\gamma_n}$, where ε_{DC} is the DC dielectric constant, ω is the frequency, and *n* is the peak number. We obtain two permittivity expressions, $\varepsilon_o(\omega)$ and $\varepsilon_e(\omega)$ by using the 0° and 90° fit parameters, respectively. The DC dielectric constant is set for each such that the index of refraction, $\mathrm{Re}\left(\sqrt{\varepsilon(\omega)}\right)$, at 0 cm^{-1}

The reference transmitted electric field is determined using the Jones matrix equations. The initial THz polarization is assumed to be in the x- direction of the lab frame. The polarization is then rotated into the reference frame of the polarizer, which lies at an angle θ relative to the initial polarization, passed through, then rotated back to the lab frame.

$$E_{ref}(\omega,\theta) = \begin{pmatrix} \cos(\theta) & \sin(\theta) \\ -\sin(\theta) & \cos(\theta) \end{pmatrix} \begin{pmatrix} 1 & 0 \\ 0 & 0 \end{pmatrix} \begin{pmatrix} \cos(\theta) & -\sin(\theta) \\ \sin(\theta) & \cos(\theta) \end{pmatrix} \begin{pmatrix} E_0 \\ 0 \end{pmatrix} = \begin{pmatrix} E_{ref,x}(\omega,\theta) \\ E_{ref,y}(\omega,\theta) \end{pmatrix}$$

The transmitted electric field through the sample and polarizer is calculated similar to the reference with the addition of the sample absorption. The THz transmitted through the polarizer is rotated into the sample frame, which has a rotation of β, through the sample, which has an absorption of $e^{i\sqrt{\varepsilon_o(\omega)}2\pi\omega d}$ and $e^{i\sqrt{\varepsilon_e(\omega)}2\pi\omega d}$, for the 0° and 90° absorption, respectively, where d is the sample thickness, d = 0.032 cm for the sample measured. The system is then rotated back to the lab frame.

$$E_{sample}(\omega,\theta,\beta)=\begin{pmatrix}\cos(\beta) & \sin(\beta)\\ -\sin(\beta) & \cos(\beta)\end{pmatrix}\begin{pmatrix}e^{i\sqrt{\varepsilon_o(\omega)}2\pi\omega d} & 0\\ 0 & e^{i\sqrt{\varepsilon_e(\omega)}2\pi\omega d}\end{pmatrix}\begin{pmatrix}\cos(\beta) & -\sin(\beta)\\ \sin(\beta) & \cos(\beta)\end{pmatrix}$$

$$\times\begin{pmatrix}\cos(\theta) & \sin(\theta)\\ -\sin(\theta) & \cos(\theta)\end{pmatrix}\begin{pmatrix}1 & 0\\ 0 & 0\end{pmatrix}\begin{pmatrix}\cos(\theta) & -\sin(\theta)\\ \sin(\theta) & \cos(\theta)\end{pmatrix}\begin{pmatrix}E_0\\ 0\end{pmatrix}=\begin{pmatrix}E_{sample,x}(\omega,\theta)\\ E_{sample,y}(\omega,\theta)\end{pmatrix}$$

The above relations are used to obtain the transmitted THz magnitude and angle of rotation. The magnitude is obtained by $\left|E_{ref}(\omega,\theta)\right|=\sqrt{E_{ref}(\omega,\theta)^T\cdot\overline{E_{ref}(\omega,\theta)}}$ and $\left|E_{sample}(\omega,\theta,\beta)\right|=\sqrt{E_{sample}(\omega,\theta,\beta)^T\cdot\overline{E_{sample}(\omega,\theta,\beta)}}$, for the reference and sample, respectively. The angle of net rotation is $\phi_{ref}(\omega,\theta,\beta)=\arctan\left(\frac{E_{ref,y}(\omega,\theta)}{E_{ref,x}(\omega,\theta)}\right)$ and

$$\phi_{sample}(\omega,\theta,\beta)=\arctan\left(\frac{E_{sample,y}(\omega,\theta)}{E_{sample,x}(\omega,\theta)}\right).$$

Due to the polarization sensitivity of the EO response, the detected THz signal is deterimined using the Planken expression:

$$\Delta I_{ref}(\omega,\theta)=\left|E_{ref}(\omega,\theta)\right|\left[\cos\left(\phi_{ref}(\omega,\theta)\right)\sin(2\varphi)+2\sin\left(\phi_{ref}(\omega,\theta)\right)\cos(2\varphi)\right]$$ and

$$\Delta I_{sample}(\omega,\theta,\beta)=\left|E_{sample}(\omega,\theta,\beta)\right|\left[\cos\left(\phi_{sample}(\omega,\theta,\beta)\right)\sin(2\varphi)+2\sin\left(\phi_{sample}(\omega,\theta,\beta)\right)\cos(2\varphi)\right]$$

[1]. Where ϕ and φ are the THz and NIR polarization angles relative to the (001) axis of a [110] cut EO crystal. The THz and NIR are incident on the [110] EO plane, perpendicular to the surface. The NIR angle φ is determined by plotting $\left|E_{ref,data}(33cm^{-1},\theta)\right|$ vs θ and $\Delta I_{ref}(33cm^{-1},\theta)$ vs θ and adjusting the NIR angle of ΔI_{ref} until there is good agreement between the data and simulation. In the case of the initial PV-CATM sucrose simulations $\varphi=43°$ gave the best agreement (see supplemental figure S2).

The change is THz signal due to the polarizer is normalized by referencing each polarizer rotation angle through $\left|T_{sample}(\omega,\theta,\beta)\right|=\frac{\Delta I_{sample}(\omega,\theta,\beta)}{\Delta I_{ref}(\omega,\theta)}$, and the relative absorption is

determined through $\Delta abs(\omega,\theta,\beta) = -2\ln\left(\frac{|T_{sample}(\omega,\theta,\beta)|}{|T_{sample}(\omega,0°,\beta)|}\right)$. This is calculated for $\omega = 0, 0.1, ..., 200$ and $\theta = 0, 15, ..., 360$ and the spectra is plotted as a surface plot as shown in the Fig. 2D. The orientation of the sucrose crystal relative to the THz polarization is unknown, so the sample orientation angle, β, is adjusted until the obtained spectra is in good agreement with the measurement. Here an orientation angle of $\beta = 62°$ is used.

[1] P. C. M. Planken, H. K. Nienhuys, H. J. Bakker, and T. Wenckebach, "Measurement and calculation of the orientation dependence of terahertz pulse detection in ZnTe," *JOURNAL OF THE OPTICAL SOCIETY OF AMERICA B-OPTICAL PHYSICS,* vol. 18, pp. 313-317, Mar 2001.
